# The Application of Glycolipid-Type Microbial Biosurfactants as Active Pharmaceutical Ingredients for the Treatment and Prevention of Cancer

**DOI:** 10.3390/ph18050676

**Published:** 2025-05-02

**Authors:** Aileen M. B. McMahon, Matthew S. Twigg, Roger Marchant, Ibrahim M. Banat

**Affiliations:** Pharmaceutical Science Research Group, Biomedical Science Research Institute, Ulster University, Coleraine BT52 1SA, UK; mcmahon-a21@ulster.ac.uk (A.M.B.M.); m.twigg@ulster.ac.uk (M.S.T.); r.marchant@ulster.ac.uk (R.M.)

**Keywords:** anticancer, microbial, biosurfactant, glycolipid, rhamnolipids, sophorolipids, mannosylerythritol lipids, trehalolipids

## Abstract

Pharmaceutical scientists have researched the potential of secondary metabolites biosynthesized by microorganisms as active pharmaceutical ingredients (APIs) for the treatment of cancer. Ideally, these APIs should possess anticancer bioactivity that specifically targets tumor cells while having little cytotoxic effect on healthy tissue. Biosurfactants are microbial secondary metabolites with surface-active properties and individual bioactivities that have the potential to either destroy cancer cells in a targeted fashion or prevent tumor cell formation. Currently, the best-studied class of microbial biosurfactants for the purpose of anticancer bioactivity is glycolipids, which contain a hydrophilic sugar moiety bonded to a hydrophobic fatty acid. Anticancer investigations are mainly carried out using in vitro models that show that compounds belonging to each of the four sub-classes of microbial glycolipid have significant anticancer bioactivity. The targeted action of this activity appears to be highly dependent on a specific congener molecular structure with nuanced alterations in structure leading to the killing of both tumor and healthy cells. This review compiles the current literature relating to glycolipid anticancer activity and provides a critical appraisal of exploiting the bioactivity of these compounds as novel anticancer agents. Finally, we propose several suggestions on how this research could be improved moving forward via method standardization.

## 1. Introduction

Microorganisms provide a rich source of naturally synthesized compounds that have the potential to be developed for pharmaceutical applications either as active pharmaceutical ingredients (API) or novel pharmaceutical adjuvants [1]. This statement is strengthened by the fact that genome sequencing has recently revealed that each microbial genome has the capacity to produce around 30–50 secondary metabolites [2]. An area where the use of microbially biosynthesized compounds is being investigated is in the treatment and prevention of cancers. Cancer is broadly defined as a group of diseases in which an abnormal change in some of the body’s cells allows them to evade apoptotic death signals whilst undergoing uncontrolled division, giving them a propensity to metastasize to healthy tissue elsewhere in the body [3]. In the UK and US, cancer is projected to account for 167,142 and 618,120 deaths in 2025, respectively [4,5]. Cancer treatment primarily involves the use of chemotherapy, radiotherapy, and surgical removal of tumors. However, chemotherapy and radiotherapy can contribute to secondary malignancy in up to 19% of patients whilst surgical intervention may not always be feasible in advanced cancers, e.g., in the case of leukemia and glioblastoma [6,7].

The screening of microbial secondary metabolites for potential new compounds that can be applied to cancer therapeutics has thus far yielded several compounds with anticancer activity, one of which is doxorubicin, an antitumor drug isolated from the soil-inhabiting bacterium *Streptomyces peucetius* [8]. Whilst doxorubicin is still a viable contemporary form of treatment for various solid cancers it has acquired an unfavorable epithet, *the red devil*, due to its association with multiple hypersensitivity reactions in patients, the most prominent of which is its impact on the cardiac system, specifically causing the onset of cardiomyopathy and subsequent congestive heart failure [9]. Adverse effects of chemotherapeutic agents reinforce the necessity for a refined approach to cancer therapy; one which produces a therapeutic capable of differentiating between healthy cells and cancerous ones. In this way, patients can benefit from a better prognosis and quality of life post treatment, effectively limiting the continuing burden on the healthcare system. In the past few years, one such group of microbial metabolites known as biosurfactants has attracted attention for their antimicrobial properties; however, these molecules are now being actively investigated for their anticancer bioactivity [10]. In this study, we collate and critically review published research that propose the use of microbial biosurfactants as novel anticancer agents. While the existing literature reports chemotherapeutic bioactivities from multiple biosurfactant classes, the glycolipid class has shown the most promise in this regard [11].

## 2. An Overview of Glycolipid-Type Biosurfactants

**Figure 1:**
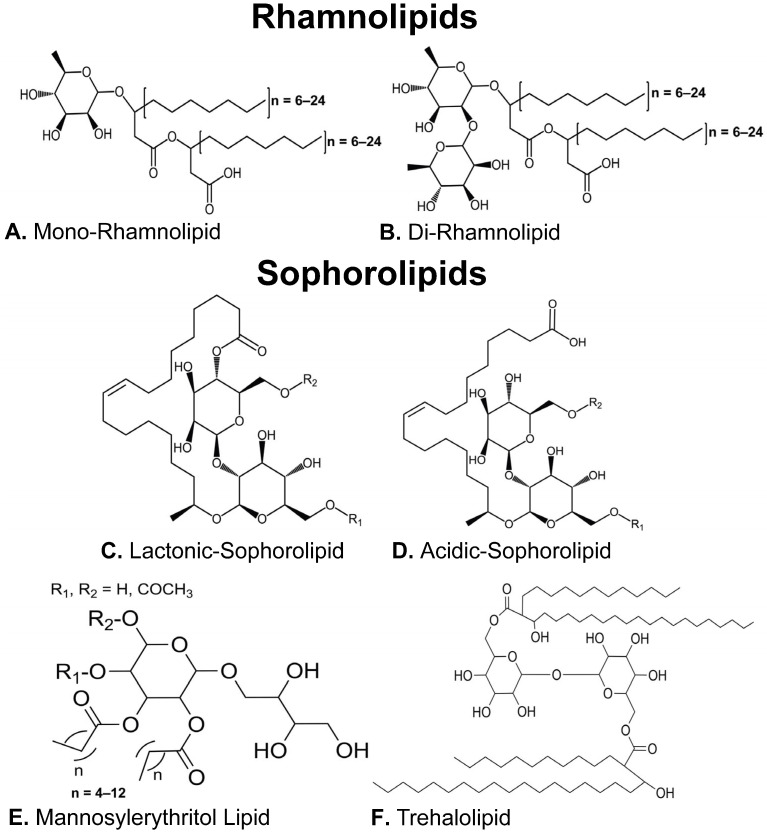
The molecular structure of each sub-class of microbial glycolipid-type biosurfactant.

Obtained from the surface of cells or secreted extracellularly by select strains of bacteria, yeast, and fungi, biosurfactants are surface-active agents capable of forming stable emulsions at the air–water or oil–water interface via reducing the surface/interfacial tensions between hydrophobic and hydrophilic structures. A key attribute of biosurfactants is their ability to form varying micelle structures. It is this physiochemical property that biosurfactant activity is most often quantified by measuring the minimum concentration of a specific biosurfactant to form micelles, a parameter referred to as Critical Micelle Concentration (CMC) [12]. Also, it is often the physiochemical properties of the biosurfactant that provide bioactivity [13]. Molecular structure differentiates biosurfactants into five major classes: the low molecular weight lipopeptides, glycolipids, phospholipids, and the high molecular weight polymeric and particulate surfactants [14]. Glycolipid biosurfactants have an amphipathic structure comprising hydrophilic carbohydrate moieties linked to hydrophobic long-chain aliphatic acids or hydroxy aliphatic acids that vary in length [15]. Variations in the hydrophilic carbohydrate moiety produce the four main distinct subclasses of microbial glycolipids: rhamnolipids, sophorolipids, mannosylerythritol lipids and trehalolipids (Figure 1) [16]. The bioactivity of biosurfactant compounds has been identified to be highly dependent on these molecular structures, specifically the hydrophobicity of that structure which is determined by the individual hydrophobic and hydrophilic moieties that make up a biosurfactant congener [13]. As to why microbial organisms synthesize biosurfactant compounds, there are several differing theories. The explanation with the most validity is that biosurfactant synthesis and export into the extracellular environment facilitates enhanced nutrient acquisition, allowing the producer organism to outcompete or out-colonize rivals in nutrient-starved environments [10,11,12,14,15].

Currently published research confirms that small molecular changes in the structure of a given glycolipid can produce large deviations in their ability to target cancer cells [13]. To appreciate the contributions of glycolipids to chemotherapy, it is essential to understand the eight key biological properties that distinguish tumorigenic cells from non-tumorigenic cells. These properties are as follows: (1) sustain proliferative signaling, (2) evade growth suppressors, (3) resist apoptosis mechanisms, (4) enable replicative immortality, (5) induce angiogenesis, (6) activate invasion and metastasis, (7) reprogram energy metabolism, and (8) evade immune destruction [17,18]. Each of the four glycolipid subclasses, to some degree, demonstrates the ability to target these cancer-associated functions.

## 3. Rhamnolipids as Anticancer Agents

Rhamnolipids (RLs) are predominantly known to be biosynthesized by bacterial species belonging to the *Pseudomonas* and *Burkholderia* genera [19]. RL congeners consist of a hydrophilic moiety comprising a methyl-pentose sugar 6-deoxy-L-mannopyranose, commonly referred to as rhamnose (conventionally contracted to Rha in congener names), conjugated via an ester linkage to a hydrophobic moiety comprising 3-(hydroxyalkanoyloxy)alkanoic acid (HAA) fatty acids. RLs may either possess a single rhamnose sugar or two rhamnose sugars and as such are classified as either mono-RL or di-RL, respectively [20]. The HAA fatty acids can either be saturated or unsaturated and vary in length between 8 and 24 carbons (Figure 1A,B) [21,22,23]. Whilst there are approximately 60 structurally distinct RL congeners, the literature reporting their anticancer activity disproportionately focuses on the four main congeners synthesized by *Pseudomonas aeruginosa* [16,19,22,24]. Interestingly the anticancer activity of rhamnolipids appears to be dependent on the structural differentiation between mono-RLs and di-RLs. This suggests that it is the hydrophilic moieties of RLs that are predominantly responsible for anticancer chemotherapeutic activity [25,26]. Whilst the results remain contradictory as to whether mono-RLs or di-RLs demonstrate superior chemotherapeutic activity, both hold merit against a range of cancer cell lines including breast, colorectal, leukemia, and skin cancer [25,26,27,28].

### 3.1. Mono-Rhamnolipids

Structurally recognizable by their single rhamnose sugar, mono-RLs are comparatively more hydrophobic than their di-RL counterparts (Figure 1A). Twigg et al. (2022) [27] postulated this increased hydrophobicity imparts greater anticancer activity against the colorectal cancer lines HCT116 and Caco-2. In this instance, in vitro cell death was induced by mono-RL, generated using a recombinant bacterial strain *P. aeruginosa* PA01 Δ*rhlC*, at concentrations as low as 10 μg mL^−1^. Cell death was observed in both colorectal cancer cell lines but not in a comparable healthy cell line control. Specifically, death occurred via necrotic pathways postulated to correspond with the mono-RL’s capacity to intercalate into the cancer cell membranes. The detrimental effects on the cancer membranes are likely initiated by the surface tension reducing properties of the mono-RL that work to destabilize membrane architecture, compromising cell integrity, resulting in death. It has been suggested that mono-RLs ability to preferentially target cancer cells is because their degree of hydrophobicity is highly compatible with the membranes of the cancer cells allowing them to adhere to exert their effects [27]. Other studies reported a different mechanism of action in which *P. aeruginosa* BN10 mono-RLs induce apoptotic/necrotic death in a panel of leukemia cell lines (HL-60, BV-173, SKW-3, and JMSU-1). This observation was in response to 25 μM mono-RLs, in which higher doses of mono-RLs were postulated to influence the overexpression of the oncogenes B-cell-lymphoma-2 (*Bcl-2*) and *c-myc* genes in BV-173 cells [25]. The relevance of this is that *Bcl-2* inhibits cell apoptosis whilst *c-myc* is a master regulator oncogene linked to increased proliferation [29,30]. Further evidence of mono-RLs ability to preferentially target cancer cells was reported by Semkova et al. (2021) [26], in which mono-RL from *P. aeruginosa* BN10 expressed this behavior against highly metastatic (MDA-MB-231) and moderately metastatic (MCF-7) breast cancer, but not in the healthy breast cancer cell line (MCF10A). Cell death was observed by a form of apoptosis known as autophagy, which appeared dose dependent in MDA-MB-231 and dose independent in MCF-7 [26]. On another occasion, mono-RLs from *P. aeruginosa* MR01 were found to heighten the expression of *p53*, a tumor suppressor gene in MCF-7 cells which occurred in the presence of 30 μg mL^−1^ mono-RL [31]. Essentially, *p53* tightly regulates glycolysis in healthy cells to operate at a threshold level to support typical cell turnover but prevent an excess of glucose to support uncontrolled cancer replication. Through increasing *p53* expression, mono-RLs act to ensure MCF-7s are deprived of excess glucose, reducing their ability to undergo unchecked cancer cell division [32,33,34]. Ultimately, mono-RLs exhibit anticancer activity via their detrimental effects on extracellular and intracellular architecture of cancer cells, whilst simultaneously displaying a capacity to interfere with cell signaling [25,27]. Encouragingly this behavior has been reported to discriminate between tumor and non-tumorigenic cells, demonstrating mono-RLs potential to circumvent the long-standing issue of non-selective chemotherapy [27].

It is believed that a moderately but not excessively hydrophobic RL structure such as a mono-RL is the most successful at adhering to the surface of cancer cells, which are characterized by a higher degree of lipid rafts (compartments rich in cholesterol and sphingolipids) than in non-cancerous cells [26]. In this way, the RL molecule can avoid collaterally damaging healthy cells in their mission to detrimentally affect cancer. As the literature shows, RL hydrophobicity can be modulated by changes in both the hydrophobic and hydrophilic portions of the structure [27]. As mono-RLs are recognized as more hydrophobic than di-RLs, further research could focus here, and whilst most research covers the effects of the 10-carbon mono-RL produced by *P. aeruginosa* (Rha-C_10_-C_10_), it is understood that fellow RL producer *Burkholdria thailandensis* produces even more hydrophobic mono-RLs with this further degree of hydrophobicity occurring due to longer fatty acid chains within the hydrophobic portion (Rha-C_12_-C_12_ to Rha-C_16_-C_16_) [35,36].

### 3.2. Di-Rhamnolipids

The increased hydrophilic di-RL structure, (Figure 1B)*,* has also been associated with anticancer bioactivity, seminal publications reporting this focus on the MCF-7 breast cancer cell line. In 2006, Thanomsub and colleagues demonstrated that the main congener synthesized by *P. aeruginosa* B189 (Rha-Rha-C_10_-C_10_) selectively inhibited the proliferation of MCF-7 cells when treated at concentrations as low as 6.25 µg mL^−1^. The authors state that no such effect was observed in a normal cell line control; however, the control cell line used, vero-cells (originally derived from a simian kidney), poses the question of whether this is a suitable control to utilize in an anticancer study focusing on human breast cancer. Whilst no specific mechanisms were revealed, Thanomsub theorized that cell death occurred due to a detergent-like effect on the cell membrane to increase permeability leading to death [37]. Similarly, in 2013, Zhao and colleagues demonstrated that the same di-RL congener, this time produced by *P. aeruginosa* M14808 induced apoptosis in the MCF-7 breast cancer cell line, and antiproliferation effects at concentrations as low as 1 µg mL^−1^. No explanation was provided for why a significantly lower concentration of Rha-Rha-C_10_-C_10_ compared to the 2006 Thanomsub study produced the same effect in this cell line [38]. Zhao et al. (2013) also go on to report similar cytotoxicity against the H460 lung cancer cell line. Interestingly, the mono-RL derivative of this congener, (Rha-C_10_-C_10_), also tested in the study exhibited no such effect on either MCF-7 or H460 [38]. A significant failing in the Zhao et al. study was that no healthy cell line control of any type was utilized in any of the anticancer assays carried out. When viewed together, these two in vitro breast cancer studies potentially indicate that di-RL mediated toxicity is affected by the specific microbial source of the RL; however, no explanation for why this is the case can be currently concluded.

The anticancer activity of di-RLs does not appear to be limited to breast cancer cells. In 2022, Adu et al. investigated the di-RL effect against the malignant melanocyte cell line SK-MEL-28, comparing activity to the non-cancerous human keratinocyte HaCaT cell line. Whilst some cytotoxicity was observed against the healthy HaCaT cells, the SK-MEL-28 melanoma cells were significantly more sensitive to di-RL treatment. SK-MEL-28 cell death occurred via necrosis in which the cell viability decreased significantly at di-RL concentrations above 40 µg mL^−1^. This process of necrotic cell death was determined to be a result of di-RL intercalation into the lipid bilayer of cells to effectively destabilize the carbon chain of the cell membrane [13]. As Callaghan et al. (2016) described, such a process works to dehydrate the phospholipid bilayer which manifests as a change in cellular adhesion and membrane functionality, eventually leading to cell death [39]. This tendency for di-RLs to exhibit greater anticancer capacity than mono-RLs was theorized to be due to the interactions between the more negatively charged functional groups on melanoma cell surfaces and the less anionic di-RLs [28]. Ultimately, this study provides a potential therapeutic route against melanoma cancer; however, the results are significantly limited by the apparent necrosis-mediated cytotoxicity against healthy skin cells as well [28].

Whilst the studies discussed above focus on how di-RLs target cancer cells by chemical and biological means, a recent study by Shen et al. (2019) [40] explores the mechanical anticancer mechanisms against the leukemia line K562 [40]. The pathological hallmark of cancer is an increase in tissue stiffness, which, if unchecked, proceeds to form a solid mass [41]. From cell line to cell line the unique optimal stiffness is controlled by the cytoskeleton, which in this study the cortical membrane tension was found to be over one-fold higher than that of neutrophils isolated from normal human blood. RLs were found to disproportionally target the stiffer leukemia cells at 20 µg mL^−1^ [40,42]. This provides an interesting context as to why di-RLs can selectively target cancer. Like mono-RLs, anticancer activity was observed to result in both necrotic and apoptotic cell death [40].

## 4. Sophorolipids as Anticancer Agents

Biosynthesized by fungi and yeast species such as *Starmerella bombicola*, sophorolipids (SLs) possess a hydrophilic head comprising a glucose disaccharide linked by a β-1,2 bond termed sophorose. The sophorose head is then in turn linked to a hydroxy fatty acid hydrophobic tail. This general structure can vary according to acetylation of the R groups located on the 6′ and/or 6″ positions of the sophorose head and the degree of saturation/length of the fatty acid chain varies between 16 and 18 carbons (Figure 1) [43,44]. SLs can be separated into two main types based on whether the carboxylic end of the fatty acid is internally esterified to generate the closed ring lactonic sophorolipid (l-SL) or free to form an acidic sophorolipids (a-SL) (Figure 1C,D) [45]. A third sub-class of SL has also been identified which possesses two sophorose moieties connected via the fatty acid, this sub-class is referred to a boliform sophorolipids (b-SL). The conversion between the two is mediated by a lactone esterase SBLE expressed from the *sble* gene which can be targeted to produce mutant strains capable of specifically producing either acidic or lactonic SL preparations [45,46,47]. Additionally, genes encoding acyl transferase enzymes are responsible for the acylation of various R groups in the SL molecule [48]. These changes in structure influence the bioactivities. The more hydrophilic acidic form is associated with higher foaming capacity and higher virucidal and pro-inflammatory cytokine activities whilst the more hydrophobic lactonic form demonstrates better surface and antimicrobial activities. Like RLs, this differentiation of structure into two main types elicits variable anticancer effects [49,50].

### 4.1. Lactonic Sophorolipids

In 2017, Li et al. postulated that the anticancer activity of SLs is directly linked to the carbon chain length and therefore the hydrophobicity of the SL congener. The more hydrophobic the SL the greater the anticancer effect. This conclusion was reached after observing the apoptotic effect of two groups of l-SLs on two cervical cancer cell lines, HeLa and CaSki. Increasing the carbon chain length from 16 to 18 correlated to an enhanced anticancer effect, evidenced by a decrease in the IC_50_ value. Importantly, this study reported that the viability of the HUVEC healthy cell line control was relatively unaffected when treated with the same congener groups. The most effective congener was the di-acetylated l-SL with a C18:1 monosaturated fatty acid chain (C18:1 Dl-SL) which was used to treat human cervical tumor xenografts in vivo with the TUNEL assay affirming C18:1 Dl-SL appeared to induce apoptosis in solid tumors [51]. At concentrations of around 30 μg mL^−1^ l-SL congeners were also found to inhibit the growth of a number of additional human cancer cell lines; H7402 and HepG2 (liver cancer cell lines); A549 (lung cancer cell line); HL-60 and K562 (leukemia cell lines); KYSE 109 and KYSE 450 (oesophageal cancer cell lines); SK-MEL-28 (melanoma cell line); and HT-29, HT115, HCT-116 and CaCo2 (Colorectal cancer cell lines) [28,39,50,52,53].

Regarding the liver cancer cell line H7402 the method by which C18:1 Dl-SL inhibited cell viability was attributed to cell apoptosis through the arrest of the cell cycle at the G1 and S phase, activation of caspase-3 and the increase of Ca^2+^ in the cytoplasm [53]. On the contrary, the a-SL derivative of this congener had a negligible anticancer effect [50]. A comprehensive study carried out by Wang et al. (2021) [52] determined the mechanisms of apoptotic death in the human liver cancer cell line HepG2 following treatment with l-SLs. It was found that the cell atrophy, chromatin condensation at the plasma membrane, and irregularities with the nucleus occurred. Additionally, l-SL was reported to interact with the membrane, leading to cell shrinkage as well as endoplasmic reticulum stress and mitochondrial vacuolation. In terms of the molecular mechanisms, it was found that l-SLs stimulated the expression of genes associated with apoptosis, including *Apaf-1*, *caspase-3, Bax*, and *Bcl-2* [52].

Cell death via apoptosis was not the only mechanism found to be mediated by l-SL. SK-Mel-28 melanoma cells treated with l-SL were shown to die via necrosis; additionally, scratch assays showed that the treatment of this cell line with l-SL prevented cellular migration, potentially prohibiting tumor cell metastasis [13]. Impressively, l-SLs show success both in vitro and in vivo to initiate apoptotic death of cancer cells; however, this is not always the case. In 2016, Callaghan and colleagues showed that a 96% pure preparation of l-SL congeners was able to inhibit the cell viability of four colorectal cancer cell lines (HT-29, HT115, HCT-116, and CaCo2), however significant decreases in the cell viability of a primary colorectal epithelia cell line (CCD-841-CoN) was also observed. Furthermore, when this l-SL preparation was administered via oral gavage to the in vivo model of colorectal cancer, the Apc^min+/−^ mouse, both gut tumor number and size increased compared to vehicle-only fed controls [39].

### 4.2. Acidic Sophorolipids

The anticancer potential of a-SLs is less understood compared to their lactonic counterparts. The efficacy of a-SLs against the human glioma cell line LN-229 was verified in which the pure acidic form induced cell death at 80 μg mL^−1^. These results are particularly impressive considering the LN-229 cell line is isolated from grade 4 glioblastoma, a form of cancer recognized for its resistance to conventional lines of therapy and incompatibility with surgical approaches. Whilst the study did not focus on the molecular mechanisms behind this cytotoxicity there is evidence that a-SLs can affect genes involved in tumor progression [54]. Twenty-four hours post treatment with a-SLs myeloma cells showed decreased mRNA expression of *PAX5*, *TLR-2*, *STAT3*, and *IL-6* at 24 h [55]. This is of interest as the dysregulation of *PAX5* transcription has been observed to facilitate the pathogenesis of lymphoma and the gene *TLR-2*, *STAT3,* and *IL-6* are associated with tumorigenesis [56].

Joshi-Navare and colleagues noted that their experimental design did not include the testing of a relevant healthy cell line control to verify that cytotoxicity is indeed selective of the cancer cell line [54]. Callaghan et al. (2022) addressed this limitation verifying that a 98% pure a-SL preparation specifically targeted colorectal cancer cell lines HCT116, HT115, HT29, and Caco-2 over the healthy colonic control cell line (CCD-841-CoN) [57]. Interestingly, this is the inverse effect observed by the same team in their previous study utilizing l-SL for targeted treatment of colorectal cancer cells [39,57]. The observed cytotoxicity was reported to simultaneously be apoptotic and necrotic in nature with the HT29 and Caco-2 cell lines being particularly susceptible; indicating that the chemotherapeutic capacity of a-SLs depends on the cell line tested. This was also the case for their 2016 l-SL study, feeding the purified a-SL to Apc^min+/−^ mice failed to cause a significant reduction in either tumor size or number. However, treatment with a-SL did seem to prevent bleeding from the inherent tumors associated with this murine model [57].

## 5. Mannosylerythritol Lipids as Anticancer Agents

Mannosylerythritol lipids (MELs) are glycolipid biosurfactants mainly produced by species belonging to the yeast genus *Pseudozyma*; however, they have also been extracted from cultures of other yeasts and some bacterial strains [58,59]. Their structure comprises a hydrophilic disaccharide moiety formed from mannose and erythritol, and a hydrophobic fatty acid and/or acetyl group (Figure 1E). In this glycolipid class, categorization is defined by the degree of acetylation of the MEL structure, of which there are four primary types: MEL-A (diacetylated), MEL-B and C (monoacetylated at the C4 and C6 positions, respectively), and MEL-D (completely deacetylated) [60]. Beyond this, changes in MELs are largely due to changes in the fatty acid chain length and its level of saturation [61]. What differentiates these glycolipids from the others is that their applications are largely focused on the cosmeceutical industry due to their good biocompatibility and recovery effects on damaged skin and hair [61]. Of the MELs tested against cancer cells, it was found that MEL-A biosynthesized by *Pseudozyma aphidis* containing C8 to C14 fatty acid chains was able to arrest murine B16F10 melanoma cells at the S phase resulting in apoptotic death [62]. Similarly, MEL-B biosynthesized by *Pseudozyma tsukubaensis* induced apoptosis in the same cell line in a dose dependent manner; however, evidence showed that the healthy cell control (NIH3T3) was also detrimentally affected [63].

In terms of human cancer cell lines, both MEL-A and MEL-B produced by *Candida antarctica* induced granulocytic differentiation of leukemic cell lines HL-60, K562, and KU812 [59]. In line with their cytotoxic effects on human cancer, Meng et al. (2024) [64] found that a MEL analog (*R*-MTL-A) was able to display selective cytotoxicity against the human skin squamous carcinoma HSC-5 cells, inducing necrosis-like death. Interestingly, this publication details an approach to design specific MEL analogs to further investigate their potential against skin cancer cells [64]. Ultimately, research to further optimize the ability of MELs to selectively target skin cancer cells is ongoing, with strategies in place to expand their reputation as anticancer agents.

## 6. Trehalolipids as Anticancer Agents

Trehalolipids (TL) are the basic component of the cell wall in *Mycobacteria* and *Corynebacteria*, they are also biosynthesized by *Rhodococcus* species [65,66,67]. TL possesses a structure comprising a non-reducing disaccharide with two glucose units connected via *α*, *α*-1, 1-glycosidic linkage [68]. Whilst the most reported TL is trehalose 6, 6′-dimycolate; an *α*-branched-chain mycolic acid esterified to the C6 position of each glucose, different structures are produced depending on the producing strain (Figure 1F) [69,70]. Whilst arguably the least investigated class of glycolipid biosurfactant regarding their anticancer activity, there is evidence they can detrimentally affect cancer cell lines in a targeted manner. This was demonstrated by Isoda et al. (1997) who reported that a succinoyl trehalolipid from *Rhodococcus erythropolis* SD-74 inhibited the growth of the human leukemia cell line U973 [59]. Essentially, the TL induced differentiation of the U973 cells into monocyte macrophages whilst remaining non-toxic to healthy human fetal lung cells [59]. Similarly, a TL isolated from *Rhodococcus wratislaviensis*, expressed cytotoxic activity against two breast cancer cell lines: MCF-7 (moderate metastatic) and MDA-MB-231 (highly metastatic) without affecting the healthy MCF10A cell line. The theorized mechanism of cell death is due to a destabilization in cell morphology characterized by alterations in the tubulin cytoskeleton, preventing mitotic spindle formation and subsequent cell division. Additionally, TLs are believed to provoke asymmetry between the inner and outer membrane bilayers, resulting in membrane invagination and eventually endosome formation. [71]. In terms of the ability of TLs to differentiate between cancerous and healthy cells, this was due to key differences between the two, namely differences in surface electric charges, surface roughness, morphology, internal microenvironment, and cytoskeleton network structures as well as differences in the phospholipid bilayer composition [72,73].

## 7. General Advantages to Utilizing Glycolipid-Type Biosurfactants as Anticancer Agents

Petro-chemical derived surfactants are widely used in chemotherapeutic formulations as excipients due to their ability to render hydrophobic APIs increasingly soluble in aqueous solutions altering the physiochemical profile of a drug to enable it to be administered to cancer cells via various routes of administration [74,75,76]. However, the use of petroleum-derived surfactants has long been associated with cytotoxicity against non-cancerous healthy tissue [77]. In comparison, glycolipid biosurfactants possess reduced toxicity and greater biocompatibility with living organisms. Perhaps the greatest advantage of microbial glycolipids is that they can combine the surface activity of surfactants to aid in drug delivery, with the bioactivity properties discussed above that enable them to potentially act as an API against cancer cells [77]. Furthermore, several in vivo studies have demonstrated that microbial glycolipids can act in a targeted fashion disrupting cancerous cell lines while having little to no effect on healthy cell line controls. This is a key advantage over many classical chemotherapeutic treatments currently in use which are highly toxic to both the cancer cells and the patient [26]. Examples of specific targeting of RL against both colorectal and breast cancers include l-SLs and a-SLs preferentially targeting cervical cancer cells and colorectal cancer cells, respectively; the MEL analog (R-MTL-A) being able to discriminate between human skin cancer and healthy skin cells; and lastly, TL preparations killed breast cancer cells but not the healthy breast cells [26,27,51,57,71]. Appreciating that chemotherapeutic agents are high-risk medications that require significant troubleshooting when determining whether they can perform their chemotherapeutic role within recommended safety procedures, the discovery of such biomolecules that express an ability to preferentially target cancer cells is promising [78].

Other than addressing the longstanding issue of hypersensitivity in patients, it is possible that glycolipid-mediated cancer therapy may be able to overcome the issue of chemoresistance increasingly encountered by conventional chemotherapeutics such as docetaxel, carboplatin, and doxorubicin. As outlined by Kar et al. (2024) [79], all three increasingly encounter the problem of chemoresistance, in which a molecular change in the tumor allows subpopulations of cells to acquire resistance to the chemotherapeutic [79,80]. In terms of docetaxel for instance it was reported that prolonged exposure to breast cancer cells led to the emergence of resistance patterns including the mTOR pathway [81]. The application of glycolipids as an anticancer agent could potentially resist this issue or be proposed as a secondary line of treatment to prevent issues with resistance. Additionally, as represented by Thakur et al. (2020), they have been reported to be easily biodegraded within the body by biological processes whilst surfactants such as transcutol-p can result in the build-up of toxic by-products in the kidneys [77]. Without intervention, such events can lead to oliguria, acute tubular necrosis, and eventually renal failure [82]. Moreover, glycolipids exhibit superior stability to surfactant counterparts, this is likely due to their microbial origin, allowing them to remain stable at extremes of salinity, temperature, and pH [83].

Recent studies have indicated that the usage of biosurfactants in large-scale applications such as household cleaning products, bioremediation, and microbial enhance oil recovery may not be economically or logistically viable [84]. More attractive applications revolve around where the bioactivity of the biosurfactant molecule(s) can be exploited at low concentrations and high price points. In the previously discussed studies, it has been established that anti-carcinogenic behavior was achieved at low concentrations [31]. Anticancer bioactivity at low concentrations reduces the amount of compound that needs to be produced via fermentation and subsequently highly purified into a chemically specific API, reducing the cost of production [84].

## 8. General Disadvantages to Utilizing Glycolipid-Type Biosurfactant as Anticancer Agents

Reports of differential anticancer bioactivity within each glycolipid subclass appear to be influenced by various factors including poor purification of glycolipid preparations, differences in producing strain, and the cancer cells being investigated [25,31,37,38]. A significant barrier to effectively establishing glycolipids as APIs in chemotherapy is that each subclass of glycolipids is produced as a heterogenous mix of congeners which need to be highly purified to be able to assign anticancer activity to a particular congener structure [22]. The issue of poor purification is perhaps the most significant for its effect on experimental results given that it makes it difficult to assign anticancer activity to a particular congener. In many cases, the dominant congener is purported to exhibit anticancer behavior, but it cannot be explicitly stated that the secondary congeners are not influential in these effects. This was discussed regarding di-RL-mediated toxicity against the moderately metastatic MCF-7 breast cancer cell line in which the *P. aeruginosa* B189 mono-RLs induced death at an MIC of 6.25 µg mL^−1^ whereas the ‘same congener’ isolated from *P. aeruginosa* M14808 cultures appeared more effective, inhibiting cells at a lower MIC value of 1 µg mL^−1^. Whilst the producing strains are different, this discrepancy was accredited to the synergistic action of secondary congeners in the *P. aeruginosa* M14808 RL preparation to enhance the detrimental effect on MCF-7 cells [37,38].

The purification process is further complicated by various factors such as the location of the molecule, ionic charge, and solubility of the compounds this procedure is time consuming to refine [85]. Compound purification is currently estimated to account for 60–80% of the total costs associated with glycolipid recovery from microbial cultures; however, as previously discussed, this cost disadvantage is somewhat negated due to the low concentration of compound(s) required for anticancer bioactivity [84]. Additionally, glycolipid recovery relies excessively on large volumes of solvents such as ethyl acetate, chloroform, methanol, pentane, and hexane all of which contribute significantly to costs but also are recognized as harmful to the environment [86]. The usage of solvent in the purification process threatens to offset the ecological benefits of using biosurfactants over chemical surfactants; further research to determine new, more eco-friendly methods of glycolipid extraction will be of great importance. Encouragingly, one such method was developed recently in 2018 for the inexpensive and environmentally friendly separation of trehalolipids from emulsified fermentation, extracting 23–58% of the total trehalolipids [87]. However, further research needs to be carried out to realize sustainable methods of large-scale production that can meet the demands of the pharmaceutical industry.

Other than costs, it is important to appreciate that the profile of glycolipid biosurfactants produced by a microbe is significantly affected by the growth conditions, including media composition and changes in pH and temperature as well as the techniques used for extraction and purification. All these factors can affect the experimental outcome, altering the distribution and yield of congeners isolated from microbial cultures [1]. In terms of MEL production from *Pseudozyma hubeiensis* changing the carbon source from olive oil to coconut oil led to a five-fold decrease in MEL-A and MEL-C production [88]. While it is possible to standardize fermentation conditions to obtain specific congeners, this variance is inconvenient should minor technological issues occur with fermentation technology. In approximately 70 publications collated and reviewed in this review, there remains to be a standardized approach to exploring the anticancer activity of glycolipid biosurfactants.

In some cases, little rationale is given for the choice of cancer cell line under study, and if a healthy cell line control is included to monitor the selective ability to target cancer cells these are often not relevant to the cancer cell they are being measured against. For instance, Isoda et al. (1997) investigated the effect of TLs against the leukemia line U973, comparing this against human fetal lung cells as the healthy cell control [59]. Another common fault in experimental design is that in many cases, a mixture of glycolipid congeners is tested against a given cancer cell line [25,31]. This makes it particularly difficult to assign a promising anticancer effect to an individual congener with many papers theorizing that differential chemotherapeutic effects could be due to the presence of a congener secondary to the dominant one identified in the mixture. Additionally, most publications focus on the anticancer effect of glycolipids in vitro which does not provide an accurate dissemination of how they interact with epithelial and endothelial barriers within living organisms, e.g., the blood–brain barrier, gastrointestinal mucous, skin, etc. For this reason, research is still in the proof-of-concept stage and will require significantly more attention before glycolipids can be realized as anticancer agents in vivo.

## 9. Perspectives for Future Work Investigating the Anticancer Activity of Glycolipids

Publications reporting the anticancer glycolipids are highly contradictory and as such future research would benefit from a more standardized investigative approach. One such flaw in the experimental design is that most publications do not provide a logistical rationale for why specific cancer cell lines are chosen for the study. Mishra et al. (2021) justified their study of the highly metastatic triple negative MDA-MB-231 breast cancer cell line due to its high mortality rate worldwide (≈200,000 new cases each year); logistically, there is no method to accurately deliver glycolipids directly to breast tissue [89]. For this reason, we believe that further research should focus on areas of the body that are more accessible to these compounds. Glycolipid preparations could be easily applied such as topical application to the skin; inhaler-mediated application to the lung and oral/ rectal application to the gastrointestinal tract [13,57,77]. An additional delivery method to be considered for future research is nanoparticle or microbubble formulations delivered to the site of the tumor loaded with the glycolipid in question alongside other drugs [90,91]. The aim of this regarding glycolipids is to weaponize their proclivity to destabilize the membrane of cancer cells, then allowing for a secondary drug in the structure to exhibit apoptotic effects. In one instance, two nanoparticles were prepared, some loaded with mono-RLs (Rha-C_10_-C_10_) and others with di-RLs (Rha-Rha-C_10_-C_10_). When compared against an in vivo xenograft mouse model bearing A431 tumors the di-RL-loaded nanoparticle specifically exhibited a higher capacity to target A431 cells, in which tumor growth was suppressed without expressing much cytotoxicity on the in vivo model [92].

A 2019 study used a similar approach of RL-loaded nanoparticles, but this time the RL was loaded alongside the hydrophobic photosensitizer ‘pheophorbide a’, prior to intravenous injection into SCC7 tumor-bearing mice. Interestingly, injection of the nanoparticles through the bloodstream resulted in high accumulation in the SCC7 tumor tissue, a murine head and neck cancer line, followed by suppression of tumor growth verified by photodynamic therapy [93]. Such findings suggest that cancer cell lines of multiple origins can be targeted via intravenous administration, at least in nanoparticle form. A poor ability to rationalize the choice of cell lines is also seen when selecting healthy cell line controls; for instance, Isoda et al. (1997) tested trehalolipids against the human leukemia cell line U973 with the effect tested against healthy human fetal lung cells [59]. Another study carried out by Joshi-Navare et al. (2011) neglects the inclusion of any healthy cell line control when testing a glioma line with SLs [54]. The absence of relevant healthy cell line controls undermines efforts to establish glycolipids as chemotherapeutic agents, limiting the conclusions that can be made to further research in the field. When included we can gain an understanding about how glycolipids may perform in vivo and assess how they confront long standing issues encountered by chemotherapy, i.e., non-selectivity.

It is important to consider ensuring the validity of results by both highly purifying and characterizing glycolipid congeners prior to investigating their anticancer bioactivity. This can be achieved by standardized techniques such as liquid and solid phase extraction [94]. Higher levels of glycolipid molecular purity can be achieved via heterologous production of specific congeners in genetically modified producer strains. Such examples include the biosynthesis of mono-RLs from a recombinant *P. aeruginosa* strain in which the *rhlC* gene encoding the enzyme that produces di-RLs was deleted or the selected production of either l-SLs or a-SLs by over expression or mutation of the lactone esterase expressing *sble* gene in *S. bombicola* [27,95]. Recombinant strains are more likely to be able to produce purer and more targeted congener structures than what can achieved from chemical manipulation of mixes derived from wild-type strains. However, glycolipid products from recombinant strains should always be analyzed as on occasion mutants generate unexpected molecular congeners as was observed recently with both acyltransferase and *sble S. bombicola* mutants, with both mutants generating high proportions of b-SL leading to a re-postulation of the SL biosynthesis pathways in *S. bombicola* altogether [96]. Regarding the chemical characterization of the glycolipid congeners prior to anticancer investigations it is suggested HPLC-MS combined with NMR is utilized. These methods, termed the ‘gold standard’ for glycolipid structural dissemination should be applied to accurately assess the effects of structural changes on the anticancer effects between glycolipid subclasses [12,97].

Appreciating that death should preferably be induced by apoptosis, researchers should amend their methodological approach to focus on measuring features of apoptosis. The process itself is characterized by morphological and biochemical changes to the internal architecture of affected cells with which there are three main pathways responsible. The first of which is initiated by death receptors on the cell surface, the second is activated by multiple stress conditions (commonly by chemotherapeutic drugs) and the third is a result of endoplasmic reticulum stress [98]. Microscopy can identify apoptotic features like cell shrinkage, cell detachment, membrane blebbing, and nuclear fragmentation whereas caspase activation can be measured using multiple caspase colorimetric kits, including the caspase-12 colorimetric assay and Caspase-Glo 3/7 assay used by Li et al. (2016) when investigating the effect of LSLs against cervical cancer [51]. The significance of measuring caspase activity is that they are a family of cysteine proteases that orchestrate the events that ensure the controlled destruction of cellular architecture and subsequent removal of apoptotic cells by phagocytes [99,100]. Another hallmark of early and late-stage apoptosis is DNA fragmentation that can be monitored via TUNEL staining in which apoptotic nuclei are positively stained to illustrate DNA fragmentation at the single cellular level [51]. However, a point to consider when observing the induction of apoptosis in cancer cells, is that it has been found that even late-stage apoptosis can be reversed by a process called anastasis, in which tumor cells expressing multiple hallmarks of apoptosis (genomic DNA breakage, caspase activation, phosphatidylserine externalization, etc.) have recovered to support tumor cell repopulation. This onset of anastasis to reverse apoptosis induced by chemotherapeutics is mediated by caspase 3-dependent secretion of prostaglandin E2, and so future approaches to detail anticancer activity of glycolipids should focus on measuring caspase 3 activity even after apoptosis has been verified by the apoptotic factors [101].

Finally, most publications document the anticancer effect of glycolipid biosurfactants in vitro, there is a gap in knowledge on what glycolipid structures can best be translated to in vivo mouse models. In terms of which glycolipids to test first, a good place to start would be RLs, SLs, or MELs because of their FDA-approved status for use on mice and rats [77]. Roberge et al. (2023) [102] report that against the breast cancer line MDA-MB-231, the IC_50_ values of a diacetylated lactonic SL vary from the 2D in vitro model from 14 µg mL^−1^ to 33 µg mL^−1^ in 3D in vivo tumor models. This suggests that without in vivo verification, it is difficult to make authentic statements about the potential of a given glycolipid to act as a novel chemotherapeutic [102].

## 10. Conclusions

**Figure 2:**
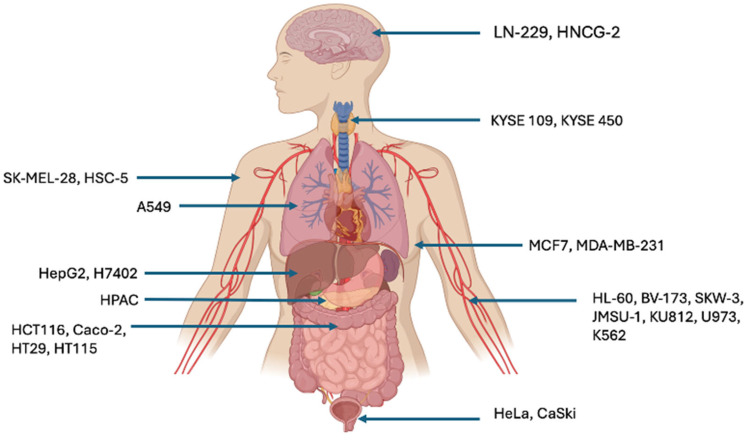
Human cancer cell lines that have been detrimentally affected by glycolipid type biosurfactant congeners and their tissue of origin (figure generated using BioRender software version 04).

This review collates publications reporting anticancer activity exhibited by each of the four main subclasses of glycolipid biosurfactants, assessing their viability to serve as novel chemotherapeutic agents. Cancer cell lines are shown to be detrimentally affected by various formulations microbial glycolipid biosurfactant, as summarized in Figure 2. Throughout, the means of cell death have been discussed, in which it has been determined that achieving apoptotic death in cancer cells is preferable [103]. However, perhaps even more important is the ability of glycolipids to preferentially target cancer cells, where each subclass demonstrated an ability to do so [26,27,64,71]. The optimization of glycolipid anticancer activity and specificity may involve the modulation or specific design of the lipid part of the molecule as the anti-carcinogenic profile of a glycolipid relies on its degree of lipophilicity [104]. This was observed in the work published by Callaghan et al. in both 2016 and 2022, who investigated SL in which a small change in the molecular structure of an SL generated a large change in their biomedical application against colorectal cancer. In this case, the more hydrophilic a-SL form which, unlike the more hydrophobic lactonic SL, did not express toxicity against healthy colorectal cells [39,57]. This influence of lipophilicity has also been observed in RLs as well; however, in this case, an increase in hydrophobicity/lipophilicity was observed to increase the resultant chemotherapeutic capacity [27]. This lipophilicity also mediates drug transport. Specifically, the ability of a molecule to permeate sheets of tightly interconnected cells typical of biological membranes is tied to lipophilicity [104]. For this reason, it can be surmised that if glycolipids are determined to induce cell death via effective permeation of the highly lipidic membranes of cancer cells in vitro, this should provide a metric on how the molecule performs in vivo. Specifically, how the glycolipid can permeate through epithelial (gastrointestinal mucous, skin) and endothelial barriers (blood–brain barriers) to successfully target the tumor of interest [104]. Ultimately, glycolipids express an ability to address multiple long-standing complications of cancer therapy. This includes the ability to selectively target cancer cells, likely reducing the onset of hypersensitivity. As well as this, glycolipids can increase the bioavailability of drugs when not acting as the API. However, much work is needed to progress to in vivo trials to fully realize the potential of these multifunctional biomolecules as anticancer agents.

## Data Availability

No new data were created or analyzed in this study. Data sharing is not applicable to this article.

## Abbreviations

The following abbreviations are used in this manuscript:

API	Active Pharmaceutical Ingredient
RL	Rhamnolipid(s)
Rha	Rhamnose
Mono-RL	Mono-rhamnolipid(s)
di-RL	Di-rhamnolipid(s)
SL	Sophorolipid(s)
l-SL	Lactonic Sophorolipid(s)
a-SL	Acidic Sophorolipid(s)
b-SL	Boliform Sophorolipid(s)
MEL	Mannosylerythritol lipid(s)
TL	Trehalolipid(s)
